# P-1214. Comparative Pharmacodynamics (PD) of Humanized Posaconazole and Isavuconazole Drug Exposures Against Wild-type and *cyp51* Mutant *Aspergillus fumigatus* Clinical Strains in the Murine Invasive Pulmonary Aspergillosis (IPA) Model

**DOI:** 10.1093/ofid/ofae631.1396

**Published:** 2025-01-29

**Authors:** Alexander Lepak, Brian D VanScoy, Catharine Vincent, Mariana Castanheira, David Andes

**Affiliations:** University of Wisconsin School of Medicine and Public Health, Madison, Wisconsin; Institute for Clinical Pharmacodynamics, Schenectady, New York; Institute for Clinical Pharmacodynamics, Schenectady, New York; JMI Laboratories, North Liberty, Iowa; University of Wisconsin, Madison, Wisconsin

## Abstract

**Background:**

Comparative pre-clinical studies examining humanized pharmacokinetic/pharmacodynamic (PK/PD) exposures of triazoles against *A. fumigatus* (AF), including clinical wild-type and c*yp51* mutants, is lacking. These studies are important to set rational clinical breakpoints, predict clinical outcomes, and provide data to guide drug and dosing choice to optimize efficacy. The aim of this study was to compare target AUC/MIC exposures in the context of expected humanized exposures for posaconazole (P) and isavuconazole (I) against AF.

Posaconazole AUC/MIC and Treatment Response in the Murine IPA Model

**Figure:**
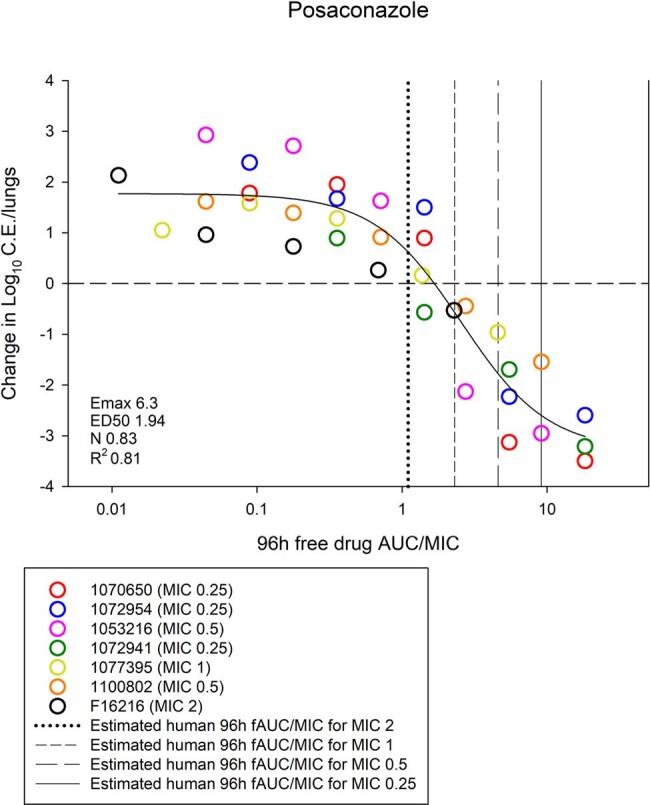

Relationship between posaconazole free drug AUC/MIC and treatment response in the animal model. The dashed horizontal line is net stasis from the start of therapy. Overlaid with vertical lines are the expected humanized AUC/MIC exposures for various MIC values (range 0.25-2 mg/L). Organisms with MIC values of ≤1 mg/L would fall in the net cidal (i.e. log kill) area on the exposure response curve based on humanized AUC/MIC exposures.

**Methods:**

A neutropenic murine model of IPA with 7 AF clinical strains (2 WT, 5 *cyp51* mutants) were utilized. MICs were determined by CLSI methods. Plasma PK were determined after single oral doses (4 dose levels) of P or I at 7 time points. Infection was induced by nasal aspiration of 50ul of a 1x10^7 conidia/ml inoculum in anesthetized mice. Treatment doses in the mouse incorporated humanized AUC exposures to examine the PK/PD relationship in the context of MIC variation within clinical strains. The duration was 96 hours. Drug efficacy was determined by qPCR of AF DNA from lyophilized lung tissue. AUC/MIC and treatment effect was modelled using the sigmoid Emax equation.

Isavuconazole AUC/MIC and Treatment Response in the Murine IPA Model

**Figure:**
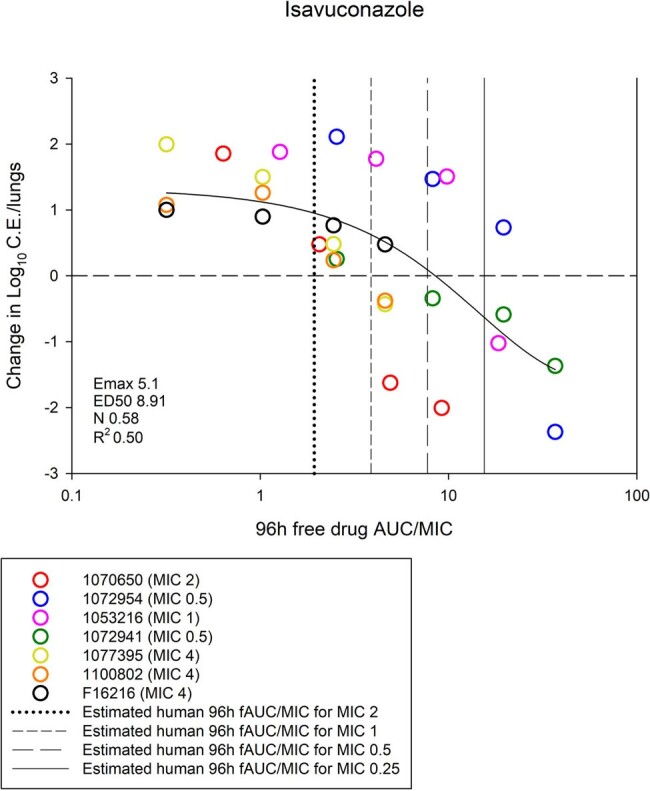

Relationship between isavuconazole free drug AUC/MIC and treatment response in the animal model. The dashed horizontal line is net stasis from the start of therapy. Overlaid with vertical lines are the expected humanized AUC/MIC exposures for various MIC values (range 0.25-2 mg/L). Organisms with MIC values of <0.5 mg/L would fall in the net cidal (i.e. log kill) area on the exposure response curve based on humanized AUC/MIC exposures.

**Results:**

P and I MIC ranged from 0.25-2 mg/L and 0.5-4 mg/L, respectively. The 96h AUC for both drugs was linear (R^2^ > 0.99). Increasing dose was associated with increased effect. A sigmoidal relationship between AUC/MIC and treatment effect was noted for both drugs. The median 96h free drug AUC/MIC target for net stasis was 1.61 for P and 4.18 for I (*P* = 0.001). The PK/PD curves for the animal model are shown in the figures. Overlaid are the expected humanized AUC/MIC exposures for various common MIC values.

**Conclusion:**

P had comparatively lower free AUC/MIC target exposures than I. Moreover, P had more potency based on expected humanized exposures. The humanized AUC/MIC exposures for P would consistently fall in the cidal activity of the efficacy curve for strains with P MIC ≤ 1 mg/L; however, I was more heterogenous and humanized AUC/MIC exposures in the cidal portion of curve occur only at MIC values < 0.5 mg/L. This data will be integrated with human clinical PK variability, MIC distributions, and clinical outcome data based on MIC for target attainment analysis and breakpoint determination.

**Disclosures:**

**Brian D. VanScoy, B.S.**, Achaogen Inc.: Grant/Research Support|Adagio Therapeutics, Inc.: Grant/Research Support|AiCurtis Anti-infective Cures AG: Grant/Research Support|Albany Medical College: Grant/Research Support|AN2 Therapeutics: Grant/Research Support|Antabio SAS: Grant/Research Support|Apogee Biologics, Inc: Grant/Research Support|Arcutis Biotherapeutics, Inc.: Grant/Research Support|B. Braun Medical Inc.: Grant/Research Support|Basilea Pharmaceutica: Grant/Research Support|BioFire Diagnostics, LLC.: Grant/Research Support|Cidara Therapeutics Inc.: Grant/Research Support|Cipla USA: Grant/Research Support|Cumberland Pharmaceuticals Inc.: Grant/Research Support|Entasis Therapeutics: Grant/Research Support|Excalibur Pharmaceuticals Inc.: Grant/Research Support|Fedora Pharmaceuticals: Grant/Research Support|Genentech: Grant/Research Support|GlaxoSmithKline: Grant/Research Support|Global Antibiotic Research and Development Partnership: Grant/Research Support|Hoffmann-La Roche: Grant/Research Support|ICPD: Employee|Inotrem: Grant/Research Support|Insmed Inc: Grant/Research Support|Iterum Therapeutics Limited: Grant/Research Support|Kaizen Bioscience: Grant/Research Support|Lassen Therapeutics Inc.: Grant/Research Support|Matinas Biopharma: Grant/Research Support|Meiji Seika Pharma Co., Ltd.: Grant/Research Support|Melinta Therapeutics: Grant/Research Support|Mutabilis: Grant/Research Support|Nabriva Therapeutics AG: Grant/Research Support|Novobiotic Pharmaceuticals LLC: Grant/Research Support|Paratek Pharmaceuticals, Inc.: Grant/Research Support|Pfizer Inc: Grant/Research Support|Praxis Precision Medicines, Inc.: Grant/Research Support|PTC Therapeutics: Grant/Research Support|PureTech LYT 100 Inc.: Grant/Research Support|Qpex Biopharma: Grant/Research Support|Renibus Therapeutics: Grant/Research Support|Sfunga Therapeutics: Grant/Research Support|Shionogi Inc.: Grant/Research Support|Spero Therapeutics: Grant/Research Support|Spruce Biosciences Inc.: Grant/Research Support|Suzhou Sinovent Pharmaceuticals Co: Grant/Research Support|Theravance: Grant/Research Support|University of Wisconsin: Grant/Research Support|US Food and Drug Administration: Grant/Research Support|UT Southwestern: Grant/Research Support|ValanBio Therapeutics, Inc.: Grant/Research Support|VenatoRx: Grant/Research Support|Zogenix International: Grant/Research Support **Catharine Vincent, Ph.D.**, Achaogen Inc.: Grant/Research Support|Adagio Therapeutics, Inc.: Grant/Research Support|AiCuris Anti-infective Cures AG: Grant/Research Support|Albany Medical College: Grant/Research Support|AN2 Therapeutics: Grant/Research Support|Antabio SAS: Grant/Research Support|Apogee Biologics, Inc.: Grant/Research Support|Arcutis Biotherapeutics, Inc.: Grant/Research Support|B. Braun Medical Inc.: Grant/Research Support|Basilea Pharmaceutica: Grant/Research Support|BioFire Diagnostics, LLC: Grant/Research Support|Cidara Therapeutics Inc.: Grant/Research Support|Cipla USA: Grant/Research Support|Cumberland Pharmaceuticals Inc.: Grant/Research Support|Entasis Therapeutics Inc.: Grant/Research Support|Excalibur Pharmaceuticals Inc.: Grant/Research Support|Fedora Pharmaceuticals: Grant/Research Support|Genetech: Grant/Research Support|GlaxoSmithKline: Grant/Research Support|Global Antibiotic Research and Development Partnership: Grant/Research Support|Hoffmann-La Roche: Grant/Research Support|Inotrem: Grant/Research Support|Insmed Inc.: Grant/Research Support|Institute for Clinical Pharmacodynamics, Inc.: Employee|Iterum Therapeutics Limited: Grant/Research Support|Kaizen Bioscience: Grant/Research Support|Lassen Therapeutics Inc.: Grant/Research Support|Matinas Biopharma: Grant/Research Support|Meiji Seika Pharma Co., Ltd.: Grant/Research Support|Melinta Therapeutics: Grant/Research Support|Mutabilis: Grant/Research Support|Nabriva Therapeutics AG: Grant/Research Support|Novobiotic Pharmaceuticals LLC.: Grant/Research Support|Paratek Pharmaceuticals, Inc.: Grant/Research Support|Pfizer Inc.: Grant/Research Support|Praxis Precision Medicines, Inc.: Grant/Research Support|PTC Therapeutics: Grant/Research Support|PureTech Health LYT 100 Inc.: Grant/Research Support|Qpex Biopharma: Grant/Research Support|Renibus Therapeutics: Grant/Research Support|Sfunga Therapeutics: Grant/Research Support|Shionogi Inc.: Grant/Research Support|Spero Therapeutics: Grant/Research Support|Spruce Biosciences Inc.: Grant/Research Support|Suzhou Sinovent Pharmaceuticals Co.: Grant/Research Support|Theravance: Grant/Research Support|University of Wisconsin: Grant/Research Support|US Food and Drug Administration: Grant/Research Support|ValanBio Therapeutics, Inc.: Grant/Research Support|VenatoRx: Grant/Research Support|Zogenix International: Grant/Research Support

